# Difficult Diagnosis of Interdigitating Dendritic Cell Sarcoma of the Retroperitoneum: A Case Report and A Brief Review of the Literature

**DOI:** 10.5146/tjpath.2026.13546

**Published:** 2026-01-31

**Authors:** Galina Boyko, Igor Makarov

**Affiliations:** Department of Pathomorphology, Almazov National Medical Research Centre, ST. Petersburg, Russia

**Keywords:** Interdigitating dendritic cell sarcoma, Retroperitoneal sarcoma, Morphological diagnosis, Differential diagnosis, Immunohistochemistry

## Abstract

Interdigitating dendritic cell sarcoma (IDCS) is a rare and aggressive neoplasm classified within the M-group of malignant histiocytoses. Its diagnosis poses a significant challenge. This article aims to describe a rare clinical case of IDCS and to illustrate the differential diagnostic process undertaken by the authors in establishing this diagnosis.

A 60-year-old woman was admitted for the resection of a retroperitoneal mass discovered via CT scan. Morphological examination revealed a 7.5×5.5×5.0 cm tumor, encapsulated by a thin fibrous capsule. The tumor was composed of 90-95% inflammatory infiltrate with lymphocyte-like cells showing mature nuclear morphology (CD3+ and CD20+ cells) mixed with histiocytes and plasma cells, and 5-10% large polymorphic spindle-shaped cells expressing expression of CD45, CD68, CD1a, CD21, CD35, CD31, and CD34. An extensive immunohistochemical panel was performed to exclude various other tumors. Based on the morphology and immunophenotype, a diagnosis of IDCS was established. Further literature analysis indicated the nonspecificity of symptoms in patients with this tumor localization and variability in CD45 and CD68 staining in tumor cells, with consistent lack of expression of CD21, CD23, CD35, CD1a, and specific T- and B-cell antigens.

IDCS is a rare and poorly understood tumor with a poor prognosis. The nonspecificity of clinical symptoms and the need for extensive morphological differential diagnosis render this entity a diagnosis of exclusion, requiring significant diligence from the pathologist.

## INTRODUCTION

Interdigitating dendritic cell sarcoma (IDCS) is an extremely rare neoplasm with an uncertain prognosis. The fifth edition of the World Health Organization’s Classification of Haematolymphoid Tumours proposes that IDCS might be included in the group of diseases related to Langerhans cells and other types of dendritic cells based on their morphological and genetic features (1). The Histiocyte Society classification has included this disease in the M group for malignant histiocytoses (2).

Clinically, IDCS typically manifests as lymphadenopathy of the cervical or axillary lymph nodes, occasionally accompanied by systemic symptoms such as weight loss, fever, fatigue, and night sweats (3). In cases of extranodal IDCS, the liver, skin, salivary glands, spleen, and tonsils are the most frequently affected sites, though systemic symptoms are uncommon in such scenarios (4). Poor prognosis is associated with younger patient age, abdominal tumor localization, and the presence of metastases.

## CASE REPORT

A 60-year-old woman with a long history of arterial hypertension on suboptimal medication, and morbid obesity (Class I), began experiencing generalized weakness, fatigue, and insomnia over the past six months. These symptoms were attributed to uncontrolled hypertension, and to exclude secondary hypertension, a CT scan of the adrenal glands and kidneys with contrast was performed. This revealed a round soft tissue mass measuring 64×62×60 mm in the left renal hilum, which showed moderate contrast enhancement (arterial phase 77 HU, venous phase 100 HU, delayed phase 77 HU). HU denotes Hounsfield units, a measure of radiodensity. For further diagnosis and surgical treatment, the patient was admitted to the Center * one month after the mass was detected.

Intraoperatively, a dense mass measuring 10×8×5 cm was found in the retroperitoneal space along the external iliac artery and the left aortic wall. The mass was located behind the left renal vein and gonadal vein, displacing the left kidney laterally. During mobilization of the mass it was discovered that the posterior wall of the mass had infiltrated the lumbar muscles. The mass was excised within the boundaries of healthy tissue with partial resection of the lumbar muscles.

**Figure 1:**
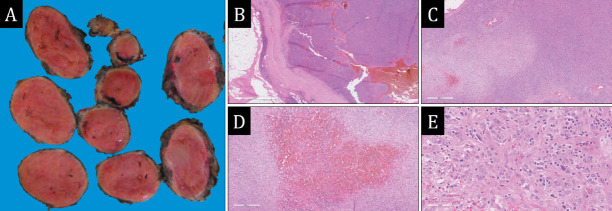
Tumor morphological study. **A)** macroscopic view of the tumor; **B)** thick-walled fibrous capsule of the tumor, beyond which a lymphocytic inflammatory infiltrate spreads, H&E, × 20; **C)** amidst a dense lymphocytic infiltrate, lighter zones represented by large spindle cells are identified, H&E, × 40; **D)** foci of hemorrhage within the structure of the spindle cell component of the tumor, H&E, × 80; **E)** histiocytic nuclear morphology of spindle cells, H&E, × 400.

Macroscopic examination of the surgical specimen revealed a nodular mass measuring 7.5×5.5×5.0 cm, surrounded by a thin fibrous capsule with surrounding adipose tissue and fragments of skeletal muscle, totaling 14.0×8.0×5.3 cm. Serial sections of the tumor showed no macroscopic evidence of capsular invasion. The tumor appeared as a light pink-yellow gelatinous mass with a glossy, bulging cut surface (Figure 1). Five lymph nodes of typical structure were found within the surrounding adipose tissue.

Microscopically, the material consisted of a lymph node with severely disturbed tissue histoarchitecture due to an infiltrate composed of 90-95% lymphocyte-like cells with mature nuclear morphology and 5-10% large pleomorphic spindle-shaped cells. The latter exhibited indistinct boundaries, scant, weakly eosinophilic cytoplasm, large pale nuclei, predominantly kidney-shaped or oval, with uneven nuclear envelope contours and 1-3 eosinophilic nucleoli. The spindle cells occasionally fused with each other via elongated cytoplasmic processes. Focal areas of coagulative necrosis (up to 5% of the tumor area) and isolated fresh hemorrhages (up to 5%) were observed. The mitotic activity of the tumor was assessed as 4 mitoses per 2 mm². The background lymphocytic infiltrate extended beyond the lymph node capsule in some areas, whereas pleomorphic spindle-shaped cells were not detected outside the capsule (Figure 1). In the surrounding adipose tissue, five lymph nodes with mild follicular hyperplasia and no signs of tumor growth were identified.

The above-described morphological findings necessitated differential diagnosis with T- and B-cell lymphomas, amelanotic melanoma, Kaposi’s sarcoma, inflammatory myofibroblastic tumor, metastatic spindle cell thymomas, Langerhans cell histiocytosis, undifferentiated pleomorphic sarcoma, malignant peripheral nerve sheath tumor, fibro-histiocytic tumors, atypical fibroxanthoma, sarcomatoid variants of renal cell carcinoma, and spindle cell variants of liposarcoma.

Immunophenotyping of the background infiltrate revealed that the lymphocyte-like cells intensely and diffusely expressed CD45 on their membranes. Among the CD45+ cells, 80-85% exhibited diffuse membrane expression of CD3, and 15-20% exhibited diffuse membrane expression of CD20. No expression of CD56 or TDT was detected in the infiltrate cells. Additionally, single infiltrate cells expressed CD1a, CD117, and CD138. The immunophenotype and mixed background infiltration with mature T and B lymphocytes reduced the likelihood of a lymphoproliferative disorder.

Amelanotic melanoma was excluded due to the absence of HMB45 and MelanA expression in tumor cells. Sarcomatoid variants of carcinomas and metastatic thymomas were excluded based on the lack of PanCK expression in tumor cells.

**Figure 2:**
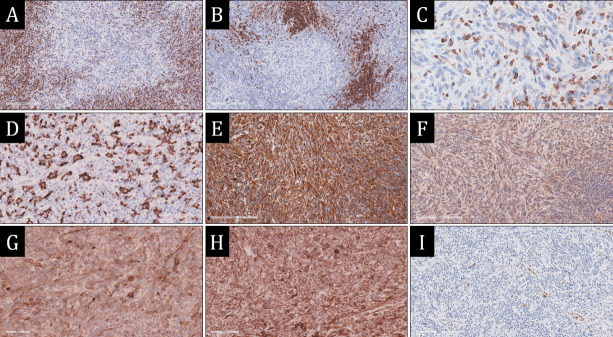
Immunohistochemical study of the tumor. **А,B)** expression of CD3 and CD20 in inflammatory infiltrate cells, × 100; **C)** absence of CD45 expression by spindle cells, with inflammatory CD45+ cells in the background of spindle cells, × 400; **D)** intense expression S100 emphasizing dendritic processes of neoplastic cells, × 800; **E, F)** expression of Vimentin and EMA by polymorphic cells, × 100; **G, H)** focal expression of CD163 and CD4 by polymorphic cells, × 400; **I)** proliferative activity in the spindle cell component as indicated by Ki67, × 200.

In large pleomorphic tumor cells, diffuse expression of vimentin, intense diffuse expression of S100 with emphasis on dendritic cell processes, uneven expression of CD168, CD4 and EMA, lack of expression of CD45, CD68, CD43, ChrA, Syn, desmin, MDM2, CDK4, D2-40, SMA, ALK, HHV-8, EBV, CD1a, CD21, CD35, SOX10, CD30, CD31, and CD34 were observed. Proliferative activity in the spindle cell component areas was 8-10% for Ki67 (Figure 2).

The low anaplasia of the tumor, combined with low proliferative activity, small foci of necrosis, and hemorrhages, made the diagnosis of undifferentiated pleomorphic sarcoma unlikely in this case. The absence of a myxoid component and the lack of SMA and ALK expression allowed the exclusion of an inflammatory myofibroblastic tumor. The growth pattern, combined with the lack of SOX10, desmin, and SMA expression, did not correspond to the morphology of a malignant peripheral nerve sheath tumor. The pronounced inflammatory infiltrate, tumor stroma characteristics, and growth pattern, combined with the lack of SMA and CD34 expression, allowed the exclusion of fibro-histiocytic tumors and fibrosarcoma. The absence of MDM2 and CDK4 expression, as well as the low anaplasia of the tumor, allowed the exclusion of liposarcoma. The lack of marked vascular proliferation and HHV-8 expression made the diagnosis of Kaposi’s sarcoma unlikely. Langerhans cell histiocytosis was excluded based on the absence of CD1a, CD68, and CD45 expression in the spindle cells. The concurrent lack of CD21, CD23, CD35, and D2-40 expression allowed the exclusion of histiocytic sarcoma and follicular dendritic cell sarcoma.

Based on the current diagnostic criteria established by the World Health Organization, including strong positive S100 staining combined with the expression of lymphohistiocytic markers (CD163 and CD4 in this case), and after an extensive differential diagnosis, a diagnosis of interdigitating dendritic cell sarcoma was made.

The postoperative period was uneventful, and a follow-up CT scan of the retroperitoneal space 6 months after tumor removal showed no evidence of recurrence or distant metastases. The patient did not receive any additional treatment and currently has a disease-free survival of 26 months.

## DISCUSSION

As demonstrated in this case, IDCS (Interdigitating Dendritic Cell Sarcoma) typically presents as a collection of pleomorphic cells, most frequently spindle-shaped. However, the literature has described various structural patterns where cells appeared ovoid or epithelioid. A singular case of IDCS reported atypical cells forming granuloma-like foci (5). These clustered cells are located in the paracortical zone of lymph nodes and sometimes infiltrate the sinuses. Interspersed among the atypical cells are T-lymphocytes and plasma cells. The nuclei are irregular and vesicular with prominent nucleoli. Occasionally, atypical mitoses, giant multinucleated cells resembling Reed-Sternberg cells, and emperipolesis can be observed (6). Areas of necrosis are rare, but the presence of diffuse necrosis is associated with a less favorable prognosis (7). The atypical cells lack Birbeck granules and intercellular junctions.

Immunohistochemical examination is crucial for the differential diagnosis of IDCS. Analysis of reported clinical cases reveals that the immunophenotype of sarcoma cells can be heterogeneous. Tumor cells consistently express the S100 protein and vimentin but variably stain for CD163, fascin, and CD45. The histiocytic marker CD68 is often focally expressed in all reported cases, although it was found in 6 out of 8 cases in the study by Xue et al. (4). Lysozyme expression was observed in most cases, although some studies reported a lack of positive staining in patients with confirmed IDCS (8). Atypical cells do not express markers of follicular dendritic cells (CD21, CD23, CD35), langerin, or specific antigens of B- and T-cells (CD20, CD3) (7). Most researchers also include CD1a in this list, although there are rare reports of CD1a-positive cells (9).

The etiology of the disease remains unclear. Whole-exome sequencing has identified 14 nonsynonymous single nucleotide polymorphisms in sarcoma cells. Particular attention is given to the POLQ gene, which encodes DNA polymerase theta, and FNIP1, a gene for folliculin-interacting protein, as their roles in malignant cell transformation have been discovered in other studies (10). Rare cases of the BRAF V600E mutation in atypical cells have also been reported (11). Di Liso E et al. reported that the use of the BRAF inhibitor vemurafenib improved patient outcomes, but the drug was later discontinued due to its toxicity (12).

Some studies highlight the potential link between B-cell neoplasms and subsequent malignant neoplasms of histiocytes and dendritic cells (13,14). For example, the t(14;18) translocation of follicular lymphoma cells was also found in histiocytic tumors that developed later. The authors suggest that altered expression of certain transcription factors or secondary acquired mutations in the MAPK pathway may explain the phenomenon of transdifferentiation of abnormal lymphoid cells into dendritic cells or histiocytes (15).

In this case, the tumor was located in the retroperitoneal space, which is atypical for IDCS, but there are descriptions in the literature with similar localization. Wang et al. describe a case of a retroperitoneal tumor in a 48-year-old patient. After surgical treatment, a course of radiotherapy was administered, but a year later, a CT scan revealed metastases in both lungs (16). Detection of IDCS in extranodal locations can be complicated by the absence of specific symptoms. In one case, two neoplasms were discovered incidentally during a medical examination: one near the left kidney (IDCS) and the other in the prostate area (leiomyosarcoma). The patient underwent radical nephrectomy without subsequent adjuvant chemotherapy, and follow-up examination a few months later showed no metastases (17). Similar localization is described by Hao et al., where the tumor was located in the right adrenal gland. After adrenalectomy, the patient chose to forgo additional chemotherapy, and a year after surgery, there were no signs of disease recurrence (18). In another case, the initial reason for seeking medical attention was upper abdominal pain, considered renal colic, but an ultrasound revealed a tumor in the left iliac fossa. Three months post-surgery, pelvic MRI showed no abnormalities (19).

More rarely, the tumor may complicate with the development of paraneoplastic syndrome. One such case is described by Jing et al., where a tumor located in the abdomen led to paraneoplastic pemphigus. A week after surgery, the patient‘s condition deteriorated, and she died of multiple organ failure (20). Zhu et al. also reported a case with an intra-abdominal tumor: a 52-year-old man presented with complaints of frequent stools and abdominal bloating. The tumor, located in the mesentery of the sigmoid colon, caused partial intestinal obstruction. Three months after surgical removal of the tumor, a CT scan revealed no recurrence or metastasis (21).

## CONCLUSION

The diagnosis of IDCS is a diagnosis of exclusion and is not possible without a comprehensive assessment of the tumor‘s morphology and immunophenotype. This requires the absence of expression of CD68, CD1a, CD21, CD23, CD35, and D2-40 in tumor cells of histiocytic morphology, along with strong positive expression of S100, emphasizing dendritic processes, and variable expression of lymphohistiocytic markers. Clinical information for some patients may be nonspecific, making the pathologist the only specialist capable of diagnosing this tumor.

## Study Limitation

CT images of the tumor are not available in this study.

## Ethical Approval

The research was approved by the local ethics committee (protocol no. 1131-22, 28.11.2022), and complied with the Helsinki Declaration. Anonymity of the patients’ and their confidentiality was preserved. This case report is written in accordance with the CARE guidelines.

## Conflict of Interest

Not declared.
